# High-dose vitamin D_3_ supplementation shows no beneficial effects on white blood cell counts, acute phase reactants, or frequency of respiratory infections

**DOI:** 10.1186/s12931-023-02642-9

**Published:** 2024-01-04

**Authors:** Gustav Wall-Gremstrup, Rune Holt, Sam Kafai Yahyavi, Mads Joon Jorsal, Anders Juul, Niels Jørgensen, Martin Blomberg Jensen

**Affiliations:** 1 Group of Skeletal, Mineral, and Gonadal Endocrinology, Department of Growth and Reproduction, Rigshospitalet, University of Copenhagen, Copenhagen, Denmark; 2 Division of Translational Endocrinology, Department of Endocrinology and Internal Medicine, Copenhagen University Hospital - Herlev and Gentofte, Copenhagen, Denmark; 3 Department of Growth and Reproduction, Copenhagen University Hospital – Rigshospitalet, Copenhagen, Denmark; 4 International Centre for Research and Research Training in Endocrine Disruption of Male Reproduction and Child Health (EDMaRC), Copenhagen University Hospital – Rigshospitalet, Copenhagen, Denmark; 5 Department of Clinical Medicine, University of Copenhagen, Copenhagen, Denmark

**Keywords:** Vitamin D, Immune system, White blood cell count, Respiratory tract infections

## Abstract

**Background:**

Vitamin D has been suggested to influence the immune system, and vitamin D metabolites and the vitamin D receptor (VDR) are generated and expressed in white blood cells (WBC). Moreover, vitamin D status has been associated with incidence and prognosis of some respiratory tract infections (RTI). Therefore, we investigated the effect of vitamin D_3_ supplementation on WBC, acute phase reactants (APR), and the risk of developing RTIs.

**Methods:**

A double-blinded, randomized, placebo-controlled clinical trial of 307 infertile men with multiple secondary immunological endpoints. The vitamin D_3_ group (*n* = 151) initially received 300,000 IU (7,500 µg) cholecalciferol once - followed by 1,400 IU (35 µg) daily for 150 days. The placebo group (*n* = 156) did not receive active ingredients.

**Results:**

At baseline, stratification into clinically relevant groups of vitamin D status (< 25; 25–50; 50–75; >75 nmol/L), showed an inverse association with total leucocyte concentrations (7.0 vs. 6.0 vs. 6.0 vs. 5.5 (10^9^/L); *p* = 0.007), lymphocytes (2.4 vs. 2.1 vs. 2.0 vs. 2.0 (10^9^/L); *p* = 0.048), CRP (2.0 vs. 1.7 vs. 1.2 vs. 1.2 (mg/L); *p* = 0.037), and orosomucoid (0.82 vs. 0.77 vs. 0.76 vs. 0.70 (g/L); *p* = 0.015). After 150 days, no differences were detected in WBC counts or APRs between the vitamin D_3_ and the placebo group. However, vitamin D_3_ treated men had a higher prevalence of self-reported RTIs compared with the placebo group (55% vs. 39%; *p* = 0.005).

**Conclusions:**

High-dose vitamin D_3_ supplementation did not alter WBCs or APRs, but a higher prevalence of respiratory infections was observed in the vitamin D_3_ group. Serum 25(OH)D_3_ was negatively correlated with most WBCs, indicating that vitamin D status may be linked with inflammation and WBC turnover, but not an important determinant of developing RTIs.

**Trial registration:**

NCT01304927 (ClinicalTrials.gov). Registered February 20, 2011.

**Supplementary Information:**

The online version contains supplementary material available at 10.1186/s12931-023-02642-9.

## Introduction

Vitamin D is a steroid hormone with several effects outside the classical regulation of bone health and calcium homeostasis [1–3]. Vitamin D is absorbed either through supplementation, diet, or de novo synthesized after ultraviolet-B radiation (UVB). Following UVB exposure of the skin, 7-dehydrocholesterol is converted into the inactive vitamin D_3_ – cholecalciferol [1]. Cholecalciferol undergoes a two-step hydroxylation before the active vitamin D metabolite 1α,25-dihydroxy-cholecalciferol (1,25(OH)_2_D_3_) binds to the vitamin D-receptor (VDR) where it can down- or upregulate genes [4]. The hepatic 25-hydroxylation enzyme CYP2R1 converts cholecalciferol into 25-hydroxy-cholecalciferol (25(OH)D_3_), which the kidneys converts into 1,25(OH)_2_D_3_, also known as calcitriol [1]. Both 25(OH)D_3_ and 1,25(OH)_2_D_3_ are catabolized into the inactive vitamin D metabolites (24,25-dihydroxyvitamin D_3_ and 1,24,25 trihydroxyvitamin D_3_, respectively) by the 24-hydroxylase enzyme CYP24A1 in the target tissue [1]. Vitamin D is mainly known for the regulation of bone and mineral homeostasis, but VDR is expressed in many other organs in the human body e.g., in the lungs, the reproductive tract, and in the immune system [2, 3, 5]. VDR is found in immune cells such as monocyte, leucocytes, and T lymphocytes [6–8]. Furthermore 1,25(OH)_2_D_3_ can be synthesized locally in monocytes as they express the enzyme CYP27B1 and vitamin D metabolites are of importance for the immune system [8].

In the innate immune system, Toll Like Receptors (TLR) exert a large role in detecting pathogenic organisms. In macrophages, activation of TLRs initiates an increase in VDR and CYP27B1 expression, and an intranuclear transcription of cathelicidin and beta-defensin genes – antimicrobial peptides [9]. Cathelicidin and beta-defensin are both present in airway epithelial cells [10] and are essential for regulating the inflammatory response in addition to eliminating the pathogens [11]. Sufficient vitamin D status is required for an adequate transcription of cathelicidin and beta-defensin [12]. Furthermore, neutrophils express VDRs and an abundant amount of cathelicidin but do not express the enzyme CYP27B1 [13]. Nonetheless, the function of vitamin D on neutrophils has not yet been fully characterized [14]. In the adaptive immune system, antigen-presenting cells (APC) present the pathogens to the naive T cells. This leads to either a proinflammatory response through Th1 (T-helper cell) development or an anti-inflammatory response through Th2 development [9, 15]. If the vitamin D levels are insufficient and if naive T cells are exposed to pathogens, Th1 development increases, which can lead to a cytokine storm where the secretion of proinflammatory cytokines IL-2 and IL-21 increases [15, 16]. Th1 will also release interferon gamma (IFN-γ) to stimulate the APC to secrete IL-6 which will activate Th17. Hence, Th17 is stimulated to secrete IL-17, another proinflammatory cytokine [17]. In contrast, sufficient vitamin D status can promote a Th2 development and IL-10 secretion and inhibit the expression of MHC-II, CD40, CD80, and CD86 in monocytes [15] and inflammatory signals influence vitamin D metabolism directly in granulomatous diseases [18].

Vitamin D deficiency is a global health issue and has been associated with inflammatory and infectious diseases [19, 20]. Vitamin D_3_ supplementation can reduce the risk of autoimmune diseases [21], and it has been suggested that impaired levels of vitamin D may negatively influence respiratory tract infections (RTI) such as asthma [22], tuberculosis [23, 24], and SARS-CoV-2 (COVID-19) [25]. Furthermore, a recent study found that COVID-19 hospitalized patients co-treated with high-dose 25(OH)D_3_ had a reduced risk of being admitted to an intensive care unit (ICU) compared to placebo treated patients [26]. However, regarding pneumonia and upper respiratory tract infections (URTI) such as the common cold and influenza, some studies suggest beneficial effects of vitamin D_3_ supplementation [27, 28], whereas other studies show no effects [29]. In this exploratory analysis from a randomized placebo-controlled clinical study, we investigated the effect of high-dose vitamin D_3_ supplementation on WBCs, acute phase reactants (APR), and self-reported RTIs.

## Materials and methods

### Trial design and intervention

The Copenhagen Bone-Gonadal Study (NCT01304927) is a single-centre, double-blinded, randomized clinical trial conducted at the Department of Growth and Reproduction, Rigshospitalet, Denmark and was approved by the Danish Health and Medicines authority, the local committee of Danish National Center for Ethics, and the data protection agency (approval no. 2010- 024588-42, H-4-2010-138, and 2,010,124,801). A thorough description of the study design has already been published [30]. In short, the study included 307 infertile men with impaired semen quality, vitamin D insufficiencies (< 50 nmol/L) at the day of screening, and no serious comorbidities. All participants were on average included in the study 2 months after the screening day. The primary endpoint was to determine if vitamin D_3_ supplementation modified the semen quality in infertile men. All participants signed a declaration of consent. The vitamin D_3_ group received an oral bolus of cholecalciferol (300,000 IU/7,500 µg) at day 1 (baseline) and vitamin D_3_ tablets containing cholecalciferol (1,400 IU/35 µg) and calcium (500 mg) for a single daily intake during each of the following 150 days. The placebo group received an oral bolus of oil and placebo tablets (without vitamin D_3_ and calcium). Written informed consent was obtained from all participants before enrolment. The treatment regime was selected to minimize the effect of compliance issues and since all men had vitamin D deficiency from the start (threshold 50 nmol/L). The study was designed to obtain an expected increase in serum 25(OH)D_3_ of 50 nmol/L. Blood samples were collected on day 1 and day 150, and participants were questioned about signs of RTIs on days 28, 90, and 150 as they were all considered incidents and therefore carefully asked for. Flow and safety outcome of the primary data concerning semen quality and changes in glucose and lipid homeostasis have been published [30, 31]. From January 2011 to August 2014 a total of 1,427 men were referred with impaired semen quality and screened for vitamin D insufficiency and comorbidities. A total of 1,002 men did not meet the criteria and were excluded, whereas 95 did not wish to participate. Furthermore, 15 men withdrew their consent and eight met an unforeseen exclusion criterion before day 1. Ultimately, 307 men were included in the study. Of the included men, 38 were lost to follow-up. In total, 269 men completed the study. No serious adverse effects were reported. The secondary outcome measures did not explicitly reference URTI. Instead, infectious disease such as pneumonia, common cold, and tonsillitis were listed as secondary outcomes. Alongside the CBG study, secondary endpoints related to metabolism [31], obesity [32], and reproductive hormones [33, 34] have been published using the dataset.

### Biochemical analysis

Leucocyte levels were conducted on a Sysmex SE9000 with a coefficient of variation (CV) of 5%. Neutrophil, eosinophil, basophil, lymphocyte, and monocyte levels on a Sysmex XE-2100 with a CV of 6%, 12%, 6%, 6%, and 15%, respectively. CRP was measured on a Cobas 8000, c702 modul with a CV of 6%, whereas orosomucoid was measured on a Cobas 8000, c502 modul with a CV of 9%. Ferritin was measured on a Modular E-modul with a CV of 7% and PTH was measured on a Cobas 8000 (Roche) with a CV of 7%. Measurements of hemoglobin (CV2%) and thrombocytes were both conducted on a Sysmex XE-2100. The vitamin D measurements of 25(OH)D_3_ (CV < 10%) and 1,25(OH)_2_D_3_ (< 18%) levels were measured with an isotope-dilution liquid chromatography-tandem mass spectrometry (LC-MS/MS).

### Statistical analysis

**Fig. 1:**
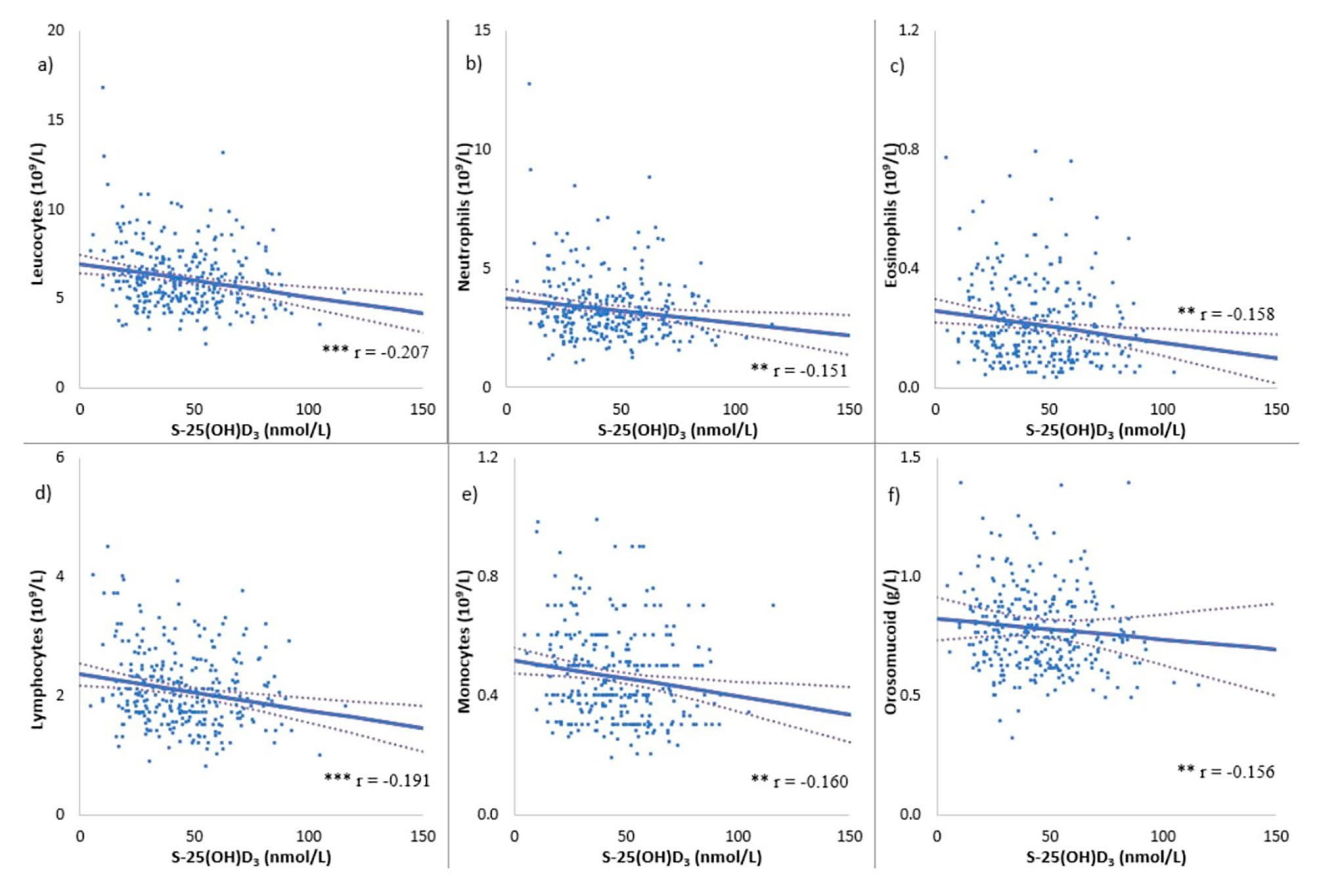
Scatterplots of inflammatory markers at baseline with 95% confidence intervals for the regression lines, as a function of serum vitamin D of all men irrespective of randomization in a pooled linear regression model. **a** Leucocytes. **b** Neutrophils. **c** Eosinophils. **d** Lymphocytes. **e** Monocytes. **f** Orosomucoid. r-values: Pearson’s correlation coefficient. *P*-values: Pearson’s correlation with a two-tailed test of significance. * *p* < 0.05, ** *p* < 0.01, and *** *p* < 0.001. Abbreviations: S-25OHD_3,_ serum 25-hydroxyvitamin D_3_

**Fig. 2:**
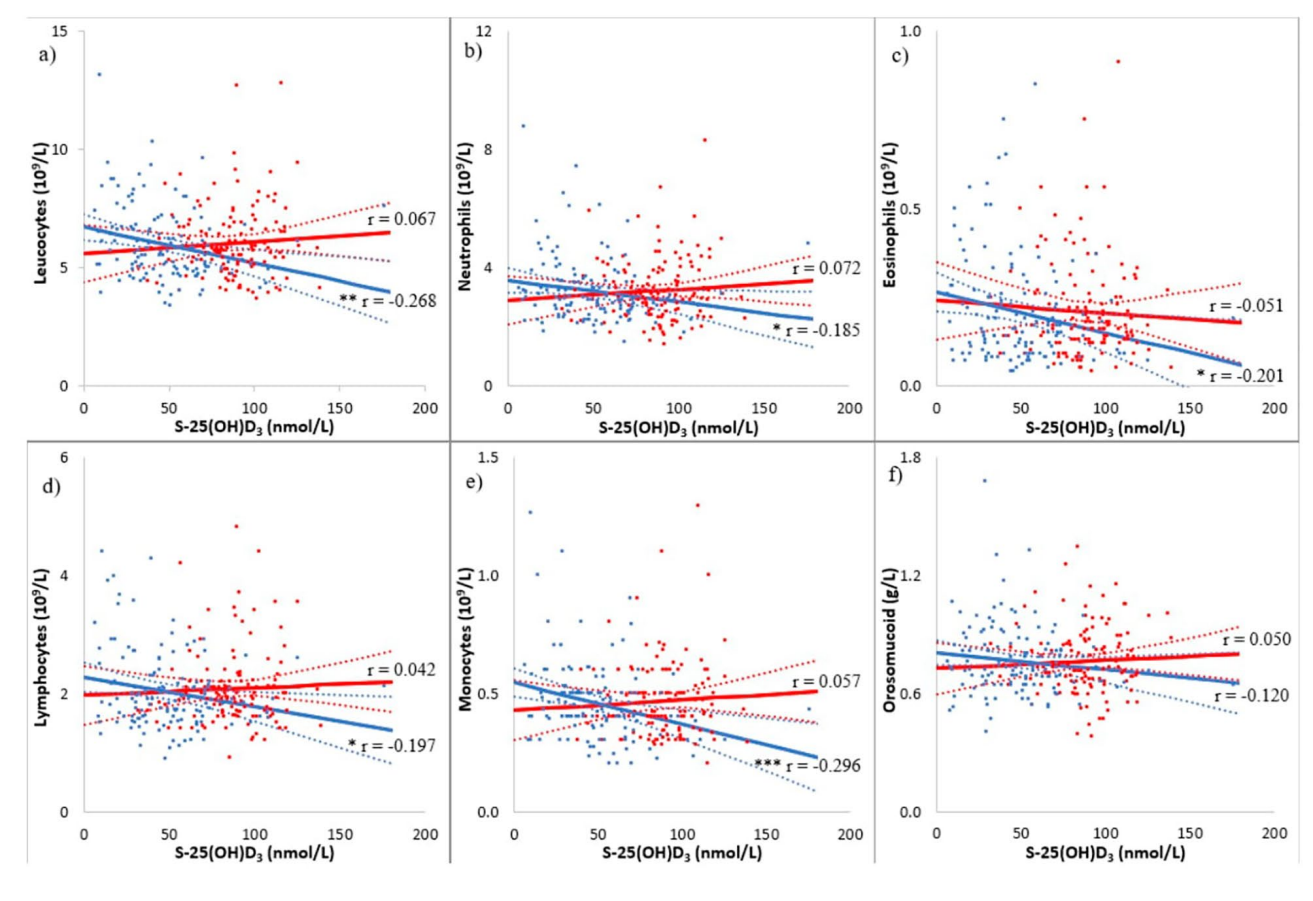
Scatterplots of inflammatory markers in the vitamin D_3_ group and placebo group after 150 days of treatment. Vitamin D_3_ group presented as red dots with red trend line and 95% confidence intervals for the regression lines. Placebo group presented as blue dots with a blue trend line and 95% confidence intervals for the regression lines, both as a function of serum 25(OH)D_3_ in a pooled linear regression model. **(a)** Leucocytes. **(b)** Neutrophils. **(c)** Eosinophils. **(d)** Lymphocytes. **(e)** Monocytes. **(f)** Orosomucoid. r-values: Pearson’s correlation coefficient. *P*-values: Pearson’s correlation with a two-tailed test of significance. * *p* < 0.05, ** *p* < 0.01, and *** *p* < 0.001. Regression *p*-values for leucocytes and monocytes were 0.016 and 0.013, respectively. Neutrophils and lymphocytes were borderline significant, 0.059 and 0.077, respectively. Regression *p*-values were calculated with Student’s t-test. Abbreviations: S-25(OH)D_3,_ serum 25-hydroxyvitamin D_3_

**Fig. 3:**
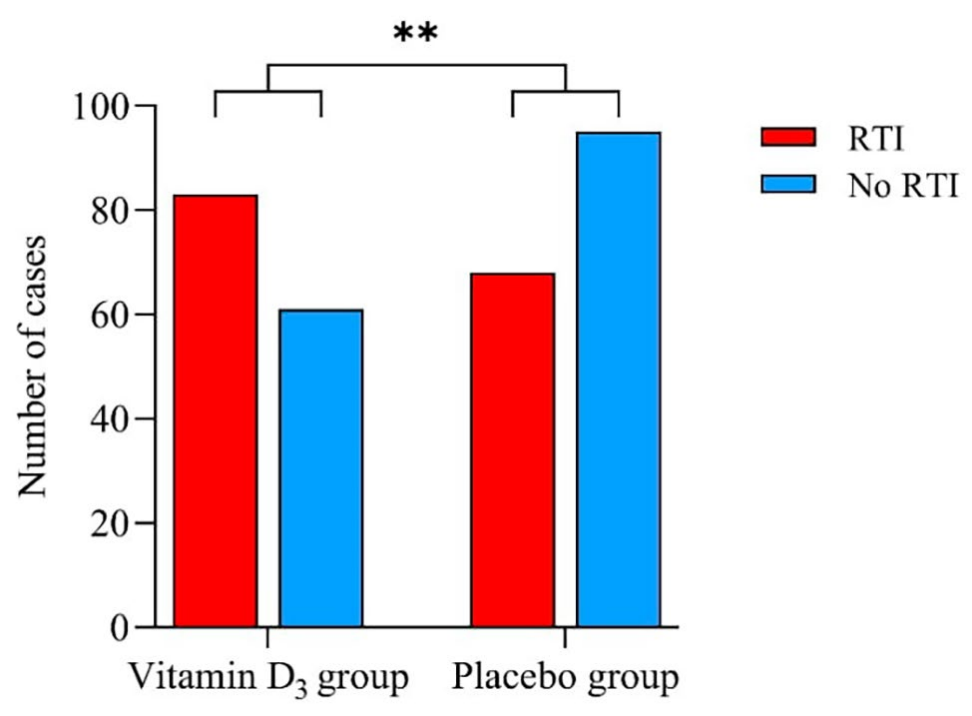
Respiratory infections after 150 days of treatment between the vitamin D_3_ group and placebo group. The data are presented as numbers of cases of RTI. The Vitamin D_3_ group had 83 cases (55%) of RTI, while 68 cases (45%) had no RTI. The placebo group had 61 cases (39%) of RTI, while 95 cases (61%) had no RTI, during the trial. Cases of common cold infections vitamin D_3_ group 61%, placebo group 45%. Influenza cases (28% and 20%, respectively). Tonsilitis cases (6% and 6%, respectively). Pneumonia (5% and 0%, respectively). **P-value: <0.01, conducted with Pearson’s Chi-squared test. Abbreviation: RTI, Respiratory tract infections

Tables 1, 2 and 3 are conducted as descriptive statistics and presented as mean with standard deviation (SD). The subgroups of vitamin D status in Table 2 are presented with *p*-values calculated with the Kruskal-Wallis test. *P*-values in Table 3 are calculated with a Student’s t-test. Furthermore, a correlation with a two-tailed test of significance and Pearson’s correlation coefficient between 25(OH)D_3_ and WBCs and APRs were completed on day 1, as seen in Fig. 1, and on day 150, as seen in Fig. 2. Moreover, Figs. 1 and 2 are presented with 95% confidence intervals for the regression lines and regression *p*-values in Fig. 2 were calculated with Student’s t-test. The self-reported RTIs (Fig. 3) were assembled as a whole number and expressed in the legend as a percentage for the vitamin D_3_ group and the placebo group. *P*-values were conducted with Pearson’s chi-squared test. All observations were included. All statistical analyses were conducted in the statistical software SPSS version 25. Vitamin D groups were defined as: Deficiency < 25 nmol/L, insufficiency 25–50 nmol/L, sufficiency 50–75 nmol/L, and adequate > 75 nmol/L.

**Table 1:** Baseline characteristics

Baseline Characteristics	*n*	Vitamin D_3_	*n*	Placebo	
Included men	151	49%	156	51%	
Age in years	151	35 (6)	156	35 (7)	
BMI	148	26.3 (4.0)	151	26.4 (4.8)	
Smokers	37	27%	31	22%	
25(OH)D_3_ (nmol/L)	147	46 (20)	153	45 (20)	
1,25(OH)_2_D_3_ (pmol/L)	146	83 (31)	151	85 (34)	
PTH (pmol/L)	117	4.5 (1.4)	116	4.9 (1.6)	
Hemoglobin (mmol/L)	150	9.3 (0.6)	150	9.3 (0.6)	
Thrombocytes (10^9^/L)	150	230 (43)	150	227 (44)	
Leucocytes (10^9^/L)	150	6.1 (1.6)	150	6.1 (2.0)	
Neutrophils (10^9^/L)	150	3.2 (1.2)	150	3.3 (1.5)	
Basophils (10^9^/L)	150	0.035 (0.021)	150	0.030 (0.019)	
Eosinophils (10^9^/L)	150	0.22 (0.13)	150	0.20 (0.15)	
Monocytes (10^9^/L)	150	0.47 (0.14)	150	0.46 (0.17)	
Lymphocytes (10^9^/L)	150	2.01 (0.64)	150	2.01 (0.62)	
CRP (mg/L)	150	1.6 (3.4)	155	1.5 (2.8)	
Ferritin (ug/L)	150	181 (112)	149	169 (93)	
Orosomucoid (g/L)	150	0.77 (0.17)	149	0.77 (0.18)	

Unless specified otherwise, the data is presented as means with Standard deviation (SD) enclosed in parenthesis. Abbreviations: BMI, body mass index; PTH, parathyroid hormone; 25(OH)D_3_, 25-hydroxyvitamin D_3_; 1,25(OH)_2_D_3_, 1,25-dihydroxyvitamin D_3_; CRP, C-reactive protein

**Table 2:** Baseline characteristics divided into four groups of vitamin D levels

Baseline characteristics	S-25(OH)D_3_; < 25 nmol/L Mean (SD)	S-25(OH)D_3_; 25–50 nmol/L Mean (SD)	S-25(OH)D_3_; 50–75 nmol/L Mean (SD)	S-25(OH)D_3_; ≥ 75 nmol/L Mean (SD)	*p*-value
Number of men, n (%)	46 (15%)	133 (44%)	96 (32%)	25 (8%)	-
Age in years	36 (7)	35 (7)	34 (7)	34 (7)	0.577
BMI	27.9 (6.6)	26.1 (4.0)	25.9 (3.7)	26.4 (4.4)	0.353
Smokers, n (%)	15 (40%)	28 (24%)	20 (22%)	3 (13%)	0.383
25(OH)D_3_ (nmol/L)	18 (5)	37 (7)	60 (7)	86 (9)	-
1,25(OH)_2_D_3_ (pmol/L)	61 (23)	74 (24)	100 (30)	120 (35)	**< 0.001**
PTH (pmol/L)	5.3 (1.6)	4.6 (1.4)	4.4 (1.4)	4.6 (1.2)	**0.020**
Hemoglobin (mmol/L)	9.4 (0.6)	9.3 (0.5)	9.2 (0.6)	9.3 (0.6)	0.051
Thrombocytes (10^9^/L)	230 (46)	225 (42)	230 (45)	233 (44)	0.720
Leucocytes (10^9^/L)	7.0 (2.5)	6.0 (1.5)	6.0 (1.7)	5.5 (1.5)	**0.007**
Neutrophils (10^9^/L)	3.8 (2.0)	3.2 (1.2)	3.3 (1.3)	2.9 (0.9)	0.068
Basophils (10^9^/L)	0.034 (0.019)	0.033 (0.022)	0.030 (0.017)	0.034 (0.024)	0.670
Eosinophils (10^9^/L)	0.25 (0.16)	0.22 (0.13)	0.20 (0.14)	0.17 (0.11)	**0.026**
Monocytes (10^9^/L)	0.51 (0.18)	0.47 (0.15)	0.44 (0.16)	0.44 (0.13)	0.063
Lymphocytes (10^9^/L)	2.4 (0.8)	2.1 (0.6)	2.0 (0.6)	2.0 (0.6)	**0.048**
CRP (mg/L)	2.0 (2.8)	1.7 (3.5)	1.2 (2.5)	1.2 (4.0)	**0.037**
Ferritin (ug/L)	180 (107)	171 (107)	176 (93)	180 (119)	0.878
Orosomucoid (g/L)	0.82 (0.18)	0.77 (0.17)	0.76 (0.16)	0.70 (0.18)	**0.015**

Unless specified otherwise, the data are presented as means with Standard deviation (SD) enclosed in parenthesis and separated into four different levels of vitamin D_3_; <25 nmol/L; 25–50 nmol/L; 50–75 nmol/L; >75 nmol/L. *P*-values: Kruskal-Wallis test. Abbreviations: BMI, body mass index; PTH, parathyroid hormone; 25(OH)D_3_, 25-hydroxyvitamin D_3_; 1,25(OH)_2_D_3_, 1,25-dihydroxyvitamin D_3_; CRP, C-reactive protein

**Table 3:** Differences between vitamin D treated and placebo treated at day 150

Day 150	*n*	Vitamin D_3_ Mean (SD)	*n*	Placebo Mean (SD)	*P*-value
25(OH)D_3_ (nmol/L)	129	89 (21)	136	51 (27)	**< 0.001**
Hemoglobin (mmol/L)	134	9.3 (0.52)	135	9.2 (0.58)	0.497
Thrombocytes (10^9^/L)	133	234 (43)	135	233 (49)	0.548
Leucocytes (10^9^/L)	133	6.0 (1.6)	135	6.0 (1.6)	0.702
Neutrophils (10^9^/L)	133	3.2 (1.1)	135	3.2 (1.1)	0.893
Basophils (10^9^/L)	133	0.036 (0.02)	135	0.033 (0.02)	0.215
Eosinophils (10^9^/L)	133	0.21 (0.14)	135	0.21 (0.15)	0.938
Monocytes (10^9^/L)	133	0.47 (0.16)	135	0.46 (0.17)	0.601
Lymphocytes (10^9^/L)	133	2.1 (0.65)	135	2.0 (0.67)	0.582
CRP (mg/L)	134	1.3 (2.1)	136	1.9 (4.8)	0.136
Ferritin (ug/L)	133	185 (121)	135	176 (102)	0.482
Orosomucoid (g/L)	132	0.76 (0.17)	135	0.77 (0.18)	0.714

The effect of treatment after 150 days. The data are presented as means with a standard deviation (SD) enclosed in parenthesis. *P*-values: Student’s t-test. Abbreviations: BMI, body mass index; 25(OH)D_3_, 25-hydroxyvitamin D_3_; CRP, C-reactive protein

## Results

### Baseline characteristics

Table 1 illustrates the baseline characteristics of the participants on day 1. On day 1, both the vitamin D_3_ and the placebo groups were on average vitamin D insufficient (< 50 nmol/L), with a serum 25(OH)D_3_ of 46 nmol/L (SD 20) and 45 nmol/L (SD 20), respectively. The concentration of leucocytes and lymphocytes, CRP, ferritin, and orosomucoid were within the reference interval with no differences between the vitamin D_3_ group and the placebo. The concentration of basophile granulocytes were higher in the vitamin D_3_ group (0.035 10^9^/L; SD 0.021) compared to the placebo group (0.030 10^9^/L; SD 0.019) on day 1 (*p* = 0.038) (data not shown).

At baseline, negative correlations were found between leucocytes (r = -0.207; *p* < 0.001), neutrophils (r = -0.151; *p* = 0.009), eosinophils (r = -0.158; *p* = 0.007), lymphocytes (r = -0.191; *p* < 0.001), monocytes (r = -0.160; *p* = 0.006), and serum 25(OH)D_3,_ (Fig. 1). Additionally, a negative correlation between orosomucoid (r = -0.156; r = 0.008) and serum 25(OH)D_3_ was found. The baseline values were divided into groups according to serum 25(OH)D_3_ levels (< 25 nmol/L; 25–50 nmol/L; 50–75 nmol/L; >75 nmol/L). Leucocytes (7.0 vs. 6.0 vs. 6.0 vs. 5.5 (10^9^/L); *p* = 0.007), eosinophils (0.25 vs. 0.22 vs. 0.20 vs. 0.17 (10^9^/L); *p* = 0.026), and lymphocytes (2.4 vs. 2.1 vs. 2.0 vs. 2.0 (10^9^/L); *p* = 0.048) were higher in men with low vitamin D status compared to men with normal/high vitamin D status (Table 2). Neutrophils (3.8 vs. 3.2 vs. 3.3 vs. 2.9 (10^9^/L); *p* = 0.068) and monocytes (0.51 vs. 0.47 vs. 0.44 vs. 0.44 (10^9^/L); *p* = 0.063) showed a similar pattern, though not significant. Additionally, CRP and orosomucoid were significantly higher in men with low vitamin D status compared to men with normal/high vitamin D status (2.0 vs. 1.7 vs. 1.2 vs. 1.2 (mg/L); *p* = 0.037) (0.82 vs. 0.77 vs. 0.76 vs. 0.70 (g/L); *p* = 0.015).

### Effect of vitamin D on WBCs, APRs and RTIs

At day 150, the concentration of 25(OH)D_3_ was higher in the vitamin D_3_ group compared to the placebo treated group. No differences in WBCs or APRs were detected between the vitamin D_3_ and the placebo group (Table 3). Moreover, no differences within the vitamin D_3_ group for WBCs or APRs were found, when comparing changes in WBCs or APRs according to vitamin D status at day 1 (Supplementary Table 1). The intervention induced an increase in serum 25(OH)D_3_ (46 to 89 nmol/L, *p* < 0.001) and 1,25(OH)_2_D_3_ levels (83 to 102 nmol/L, *p* < 0.001) in the vitamin D_3_ group that differed from the placebo group. There was no significant correlation between serum 25(OH)D_3_ and WBCs or APRs in the vitamin D_3_ treated group at day 150, as seen in Fig. 2. We did establish a significant difference of the regression lines between the two groups for leucocytes (*p* = 0.016) and monocytes (0.013), and borderline significant for neutrophils (*p* = 0.059) and lymphocytes (*p* = 0.077). In the placebo group, there was a significant correlation between serum 25(OH)D_3_ and leucocytes (r = -0.268, *p* = 0.002), neutrophils (r = -0.185, *p* = 0.033), eosinophils (r = -0.201, *p* = 0.021), lymphocytes (r = -0.197, *p* = 0.023), monocytes (r = -0.296, *p* < 0.001) at day 150, as seen in Fig. 2. In the vitamin D_3_ group, 83 participants reported at least one episode of a RTI corresponding to 55% of all men in the vitamin D_3_ group. Noteworthy, men in the vitamin D_3_ treated group reported more respiratory infections (common cold, influenza virus, tonsilitis, and pneumonia) during the trial period compared to the placebo group (61 participants, 39% of placebo group; *p* = 0.005), (Fig. 3). The etiological factors contributing to RTIs were common cold and influenza virus based on questionnaire, but not confirmed by serological testing. In men, who had self-reported RTIs in the vitamin D_3_ treated group, the common cold was reported by 61% (51 participants) whereas in the placebo group, it was reported by 74% (45 participants). Furthermore, 28% (23 participants) of the RTIs in the vitamin D_3_ treated group and 20% (12 participants) of RTIs in the placebo group reported influenza. Baseline characteristics such as smoking status, age, and BMI were compared to cases of RTIs between the vitamin D_3_ group and placebo group and showed no significant differences (data not shown).

## Discussion

This study shows no significant effects of vitamin D_3_ supplementation on white blood cells (WBC) or acute phase reactants (APR) in infertile men. Although at baseline negative associations between serum 25(OH)D_3_ and WBCs and orosomucoid were found. Moreover, men with low vitamin D status (< 25 nmol/L; 25–50 nmol/L) had significantly higher levels of WBCs and APRs compared to normal/high vitamin D status (50–75 nmol/L; >75 nmol/L). After 150 days of intervention, there was a significantly higher prevalence of RTIs in the vitamin D_3_ group compared to the placebo group despite no differences in WBCs. The existing literature on how vitamin D insufficiency affects WBCs and APRs, as well as URTIs and pneumonia in young men, who despite infertility can be considered, is limited. The negative associations with leucocytes, neutrophils, eosinophils, monocytes, lymphocytes, and orosomucoid are similar to the study by Laird et al. [35] who investigated the link between vitamin D status and inflammatory markers such as CRP and IL-6 in an elderly population (> 60 years of age). They discovered significant associations in CRP and IL-6 levels compared to vitamin D levels. Specifically, individuals with lower levels of serum vitamin D had higher levels of CRP and IL-6, though within reference levels. The significant negative correlations found in both studies suggest that the link with inflammation is not age dependent. At day 150, we found a similar negative correlation between serum 25(OH)D_3_ and the inflammatory markers in the placebo group (Fig. 2), but no significant correlation in the vitamin D_3_ group. This could indicate that vitamin D influences inflammatory markers, even when within reference levels, but vitamin D_3_ supplementation and vitamin D status are not strong determinants of the risk of acquiring a RTI. Vitamin D has been the subject of discussions regarding a potential U-shaped pattern concerning health risks and immune function [36, 37]. The U-shaped relationship of vitamin D suggests that vitamin D_3_ supplementation may only influence inflammatory markers up to a certain threshold. This could potentially explain why no further effects in the vitamin D_3_ group were observed after 150 days. Moreover, it is possible that the high-dose bolus of 300,000 IU is the cause of the potential harmful effect on the incidence of RTI.

The lack of benefit of high dose vitamin D_3_ supplementation on RTIs was consistent with other studies [38, 39]. Subsequently, more recent research has found contradictory results. Several studies using bolus dosing such as the ViDA study [40] with monthly doses of 100,000 IU showed no benefit for respiratory infections, while 60,000 IU monthly showed similar results but reduced the burden of symptoms [41, 42]. A recent meta-analysis [43] showed a 20% reduction in RTI in patients receiving daily or weekly dosing, but not in patients treated with bolus dosing.

Laaksi et al. [44] and Urashima et al. [45] showed that participants who ingested daily low-dose vitamin D_3_ (400 IU and 1,200 IU, respectively) had a positive effect on URTI. Mechanistic insight into the difference between high bolus versus daily intake was provided by Vieth et al. [46] suggesting that high-dose vitamin D_3_ intake could induce substantial fluctuations in the concentration of serum 25(OH)D_3_, differently than daily low-dose vitamin D_3_ intake, through an imbalance in the vitamin D regulating enzymes (CYP27B1 and CYP24A1). This results in a decreased level of active vitamin D (1,25(OH)_2_D_3_) needed to assist the immune system against pathogens in respiratory infections and in this way, high-dose bolus vitamin D may impair immune function.

It is plausible that vitamin D insufficiency is of less relevance in milder infections, particularly if the person does not have vitamin D deficiency but may play a more essential role when exposed to severe respiratory tract infections. This hypothesis is supported by a study from Castillo et al. [26], which showed that high-dose 25(OH)D_3_ supplementation was associated with reduced intensive care unit (ICU) cases in SARS-CoV-2 hospitalized patients. Additionally, a study from Nielsen et al. [25] found that vitamin D deficiency was significantly correlated with severe SARS-CoV-2 infections. Nonetheless, Nielsen et al. [25] also found significant differences between vitamin D and age as well as vitamin D and comorbidity, which may explain why we did not observe any differences between vitamin D insufficiency and inflammatory markers or for that matter URTI. Except for their known infertility, our study participants were young and healthy, and none were hospitalized during the trial. But hypothetically, in the event participants had been hospitalized with a RTI during the trial, the results would likely have been akin.

The significant difference in respiratory infections in the vitamin D_3_ group compared to the placebo group, 55% vs. 39% cannot be explained by this study setup. Moreover, URTIs were not a predefined secondary endpoint, and this observation should therefore be considered as an explorative endpoint that needs confirmation in future trials. Furthermore, the CBG (Copenhagen Bone-Gonadal Study) has several secondary endpoints, which is an important limitation because the likelihood of chance finding increases with numerous secondary endpoints. To our knowledge, no studies have shown an increased prevalence of RTIs and vitamin D_3_ supplementation. This could be due to the self-reported procedure following interview for potential incidents during GCP (Good clinical practice) monitored follow up. No tests were done when participants reported RTIs over the 150 days to confirm the diagnosis. On average, healthy men experience a URTI 1–2 times a year [47], which correlates with the cohort, as 46% of the participants experienced a URTI within 150 days. From the respiratory infections, the vitamin D_3_ group had a higher percentage (5%) of pneumonia cases compared to the placebo group (0%). However, pneumonia is a lower respiratory tract infection. Similarly, to our study, Remmelts et al. [48] explored three case-control studies of a total of 33,726 cases of pneumonia and found that vitamin D_3_ supplementation in adults showed no preventive effect against pneumonia when adjusted for confounders. One of the studies even reported an increased risk of pneumonia in the vitamin D_3_ group. When exploring respiratory infections, we found that vitamin D_3_ did not protect against influenza. Influenza was detected in 28% of the RTIs in the vitamin D_3_ group, whereas 20% were in the placebo group. We assume that high-dose vitamin D_3_ intake does not influence mild, non-hospitalized URTI, as opposed to low-dose vitamin D_3_ supplementation.

This study has notable strengths. All participants were vitamin D insufficient at the day of screening, and the serum 25(OH)D_3_ was analysed on a LC-MS/MS. Additionally, participants in the vitamin D_3_ group achieved the expected increase in serum 25(OH)D_3_ after 150 days [30]. However, while valid, it is not without limitations. We investigated 307 men, who were healthy besides being infertile and having vitamin D insufficiencies. This population may not be suitable to represent healthy men in general. Furthermore, after 150 days of intervention, the placebo group had an average vitamin D status above 50 nmol/L. This observation raises the possibility that the outcome of WBC and APR levels between the vitamin D_3_ group and placebo group may be influenced by inclination. In retrospect, using a higher daily dosage rather than the initial bolus of 300,000 IU would have been preferable. The decision to opt for the initial bolus of 300,000 IU was driven by concerns about non-compliance within the vitamin D_3_ group, potentially resulting in negligible differences in the vitamin D status between the two groups. While the chosen dosage regimen guaranteed a pronounced divergence in vitamin D status, the utility of the initial 300,000 IU bolus remains uncertain, introducing the possibility of underestimating the positive effects associated with correcting vitamin D insufficiency. The high bolus dosage has later shown to induce harm and may lead to high CYP24A1 activity in many tissues.

In conclusion, this exploratory analysis from a randomized clinical trial did not show any impact of vitamin D_3_ supplementation in infertile men regarding WBCs and APRs. However, serum 25(OH)D_3_ was significantly negative correlated with leucocytes, neutrophils, eosinophils, monocytes lymphocytes, and orosomucoid at baseline. In our cohort, vitamin D_3_ has no preventive effect against non-hospital required RTIs and high-dose vitamin D_3_ supplementation cannot be routinely recommended for individuals with insufficient vitamin D levels to prevent respiratory tract infections.

### Electronic supplementary material

Below is the link to the electronic supplementary material.

**Supplementary Material 1**: **Table 1** Effect of treatment as Δ values divided into four groups of vitamin D levels. The effect of treatment presented as delta values after 150 days divided into four groups of vitamin D_3_ levels from day 1; <25 nmol/L; 25–50 nmol/L; 50–75 nmol/L; >75 nmol/L. The *n* highlights the number of participants in each group. The data are presented as means with a confidence interval of ± 95% (CI 95%) enclosed in parenthesis. *P*-values: t-test. Abbreviations: BMI, body mass index; 25(OH)D_3_, 25-hydroxyvitamin D_3_; CRP, C-reactive protein. **Table 2** Season of inclusion. The season of inclusion during the trial. 46% of participants were included during the season of spring, 19% during summer, 10% during autumn, and 26% during winter.

## Data Availability

The datasets used and analysed during the current study are available from the corresponding author on reasonable request.

## Abbreviations

1,25(OH)_2_D_3_: 1,25-dihydroxyvitamin D3
25(OH)D_3_: 25-hydroxyvitamin D_3_
APC: Antigen presenting cell
APR: Acute phase reactant
BMI: Body mass index
CI: Confidence interval
CRP: C-reactive protein
CV: Coefficient of variation
ICU: Intensive care unit
IFN-γ: Interferon gamma
PTH: Parathyroid hormone
RTI: Respiratory tract infection
Th1: T-helper cell 1
Th2: T-helper cell 2
TLR: Toll like receptor
URTI: Upper respiratory tract infections
UVB: Ultraviolet-B
VDR: Vitamin D receptor
WBC: White blood cell
