# Supplementary material for: High-dose vitamin D3 supplementation shows no beneficial effects on white blood cell counts, acute phase reactants, or frequency of respiratory infections
Source: Respir Res. 2024 Jan 4;25:11. doi: 10.1186/s12931-023-02642-9 (PMC10765571; doi:10.1186/s12931-023-02642-9)
Supplement: Supplementary file 1 — Supplementary Material 1: Table 1 Effect of treatment as Δ values divided into four groups of vitamin D levels. The effect of treatment presented as delta values after 150 days divided into four groups of vitamin D3 levels from day 1; <25 nmol/L; 25–50 nmol/L; 50–75 nmol/L; >75 nmol/L. The n highlights the number of participants in each group. The data are presented as means with a confidence interval of ± 95% (CI 95%) enclosed in parenthesis. P-values: t-test. Abbreviations: BMI, body mass index; 25(OH)D3, 25-hydroxyvitamin D3; CRP, C-reactive protein. Table 2 Season of inclusion. The season of inclusion during the trial. 46% of participants were included during the season of spring, 19% during summer, 10% during autumn, and 26% during winter. [file 12931_2023_2642_MOESM1_ESM.docx]

## **Supplementary**

***Table 1*** *– Effect of treatment as Δ values divided into four groups of vitamin D levels:
(next page)*

| **Effect of treatment**  **in subgroups between day 1**  **and day 150** | **Vitamin D_3_ group** | **Placebo group** |  | **Vitamin D_3_ group** | **Placebo group** |  | **Vitamin D_3_ group** | **Placebo group** |  | **Vitamin D_3_ group** | **Placebo group** |  |
| --- | --- | --- | --- | --- | --- | --- | --- | --- | --- | --- | --- | --- |
|  | Vitamin D_3_  < 25 nmol/L  *n* = 16  Δ mean  (Cl95%) | Vitamin D  < 25 nmol/L  *n* = 22  Δ mean  (Cl95%) | p-value | Vitamin D  25-50 nmol/L  *n* = 58  Δ mean  (Cl95%) | Vitamin D_3_  25-50 nmol/L  *n* = 54  Δ mean  (Cl95%) | p-value | Vitamin D_3_  50-75 nmol/L  *n* = 40  Δ mean  (Cl95%) | Vitamin D_3_  50-75 nmol/L  *n* = 48  Δ mean  (Cl95%) | p-value | Vitamin D_3_  ≥ 75 nmol/L  *n* = 11  Δ mean  (Cl95%) | Vitamin D_3_  ≥ 75 nmol/L  *n* = 10  Δ mean  (Cl95%) | p-value |
| Δ 25(OH)D_3_  (nmol/L) | 68.5  (56.8 - 80.2) | 21.5  (10.8 - 32.1) | **<0.001** | 51.3  (44.4 - 58.2) | 16.1  (9.6 - 22.7) | **<0.001** | 26.6  (21.6 - 31.6) | -4.8  (-13.5 - 3.8) | **<0.001** | 8.4  (-4.9 - 21.8) | -40.0  (-54.4 - -25.5) | **<0.001** |
| Δ Hemoglobin (mmol/L) | -0.153  (-0.340 - 0.037) | -0.229  (-0.380 - -0.080) | 0.541 | 0.003  (-0.084 - 0.090) | 0.023  (-0.070 - 0.100) | 0.753 | 0.107  (-0.001 - 0.220) | -0.006  (-0.130 - 0.120) | 0.187 | 0.018  (-0.280 - 0.320) | -0.150  (-0.390 - 0.090) | 0.397 |
| Δ Thrombocytes  (10^9^/L) | 8.6  (-9.3 - 26.5) | 5.6  (-1.6 - 12.8) | 0.725 | 3.8  (-2.9 - 10.4) | 7.2  (2.4 - 11.8) | 0.424 | 3.6  (-4.1 - 11.3) | 0.4 (-8.2 - 9.0) | 0.590 | 10.2  (1.6 - 18.7) | 2.6  (-8.4 - 13.6) | 0.286 |
| Δ Leucocytes  (10^9^/L) | 0.147  (-0.46 - 0.75) | -0.063  (-0.65 - 0.53) | 0.639 | -0.079  (-0.43 - 0.27) | -0.035  (-0.35 - 0.28) | 0.854 | 0.017  (-0.45 - 0.49) | -0.104  (-0.55 - 0.34) | 0.707 | 0.164  -0.66 - 0.98) | -0.030  (-0.39 - 0.33) | 0.682 |
| Δ Neutrophils  (10^9^/L) | 0.269  (-0.201 - 0.739) | 0.063  (-0.415 - 0.541) | 0.565 | -0.054  (-0.358 - 0.250) | -0.079  (-0.350 - 0.193) | 0.904 | 0.007  (-0.429 - 0.442) | -0.111  (-0484 - 0.263) | 0.682 | 0.045  (-0.512 - 0.601) | -0.100  (-0.382 - 0.182) | 0.658 |
| Δ Basophils  (10^9^/L) | 0.003  (-0.006 - 0.013) | 0.004  (-0.001 - 0.010) | 0.873 | 0.0002  (-0.005 - 0.006) | 0.003  (-0.001 - 0.006) | 0.498 | 0.003  (0.003 - 0.002) | 0.002  (-0.001 - 0.005) | 0.649 | 0.01  (0.001 - 0.019) | 0.003  (-0.007 - 0.013) | 0.306 |
| Δ Eosinophils  (10^9^/L) | -0.032  (-0.07 - 0.005) | 0.007  (-0.024 - 0.038) | 0.116 | -0.002  (-0.027 - 0.022) | 0.012  (-0.011 - 0.035) | 0.399 | 0.002  (-0.031 - 0.036) | 0.004  (-0.018 - 0.027) | 0.927 | 0.003  (-0.05 - 0.06) | 0.022  (-0.04 - 0.081) | 0.636 |
| Δ Monocytes  (10^9^/L) | -0.006  (-0.059 - 0.047) | 0.031  (-0.043 - 0.106) | 0.477 | -0.008  (-0.043 - 0.027) | 0.001  (-0.031 - 0.033) | 0.711 | 0.011  (-0.024 - 0.045) | -0.003  (-0.043 - 0.037) | 0.609 | 0.044  (-0.030 - 0.117) | 0.000  (-0.077 - 0.077) | 0.421 |
| Δ Lymphocytes  (10^9^/L) | -0.093  (-0.38 - 0.19) | -0.165  (-0.29 - -0.04) | 0.604 | -0.013  (-0.105 - 0.078) | 0.030  (-0.083 - 0.142) | 0.552 | 0.023  (-0.147 - 0.193) | 0.004  -0.100 - 0.109) | 0.848 | 0.077  (-0.164 - 0.319) | 0.011  (-0.226 - 0.248) | 0.700 |
| Δ CRP  (mg/L) | 0.53  (-0.4 - 1.4) | 1.60  (-0.7 - 3.9) | 0.484 | 0.57  (-1.3 - 0.2) | -0.27  (-1.1 - 0.5) | 0.586 | -0.29  (-1.2 - 0.64) | 0.68  (-1.1 - 2.5) | 0.357 | 0.00  (-0.3 - 0.3) | -1.6  (-4.7 - 1.7) | 0.318 |
| Δ Ferritin  (ug/L) | 7.9  (-24 - 40) | 7.8  (-8 - 23) | 0.993 | -0.6  (-12 - 10) | 1.8  (-8 - 23) | 0.741 | 9.4  (-6 - 25) | 0.9  (-14 - 16) | 0.429 | 5.4  (-8 - 19) | 9.3  (-4 - 22) | 0.681 |
| Δ Orosomucoid  (g/L) | 0.015  (-0.121 - 0.151) | 0.026  (-0.041 - 0.092) | 0.873 | -0.019  (-0.053 - 0.016) | -0.013  (-0.044 - 0.019) | 0.801 | 0.014  (-0.031 - 0.059) | -0.004  (-0.065 - 0.057) | 0.646 | 0.000  (-0.063 - 0.063) | -0.029  (-0.144 - 0.086) | 0.656 |

The effect of treatment presented as delta values after 150 days divided into four groups of vitamin D_3_ levels from day 1; <25 nmol/L; 25-50 nmol/L; 50-75 nmol/L; >75 nmol/L. The *n* highlights the number of participants in each group. The data are presented as means with a confidence interval of ±95% (CI 95%) enclosed in parenthesis. P-values: t-test. Abbreviations: BMI, body mass index; 25(OH)D_3_, 25-hydroxyvitamin D_3_; CRP, C-reactive protein.

***Table 2 –*** *season of inclusion:*
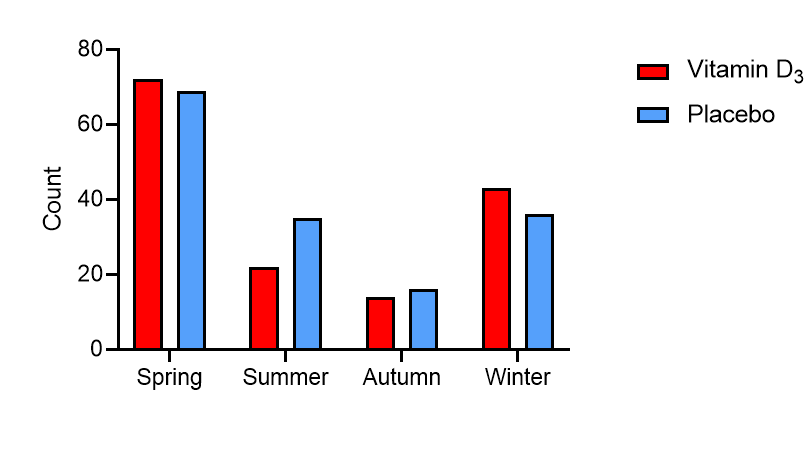


The season of inclusion during the trial. 46% of participants were included during the season of spring, 19% during summer, 10% during autumn, and 26% during winter.
